# Are saltmarshes younger than mangrove swamps?

**DOI:** 10.1002/ece3.8481

**Published:** 2022-01-11

**Authors:** Geerat J. Vermeij

**Affiliations:** ^1^ Department of Earth and Planetary Sciences University of California‐Davis California USA

**Keywords:** Halophyte, Mangrove, Paleoecology, Saltmarsh

## Abstract

Temperate saltmarshes and tropical mangrove swamps (mangals) are marine‐influenced, productive ecosystems that enhance nutrient transfers between land and sea and facilitate colonization of lineages between terrestrial and marine habitats. Mangals have existed since the late Cretaceous, but the time of origin of saltmarshes is less clear. On the basis of phylogenetic and fossil evidence for plants and molluscs specialized to these ecosystems, I propose that saltmarsh vegetation of angiosperms began during the latest Eocene to Early Oligocene (35–30 Ma), at least 34 m.y. after the origin of mangals. The plants that colonized saltmarshes then and later have mainly temperate origins, contrasting with the tropical‐forest origins of mangroves. Unlike the plants, the few saltmarsh‐specialized molluscs are derived from tropical lineages and reflect recent colonizations. The development of saltmarshes during the Neogene enhanced near shore productivity along temperate and Arctic coastlines.

## Abstract 2

Saltmarsh vegetation originated at least 35 million years after angiosperm mangroves.

## 1 INTRODUCTION

Temperate saltmarshes and tropical mangrove swamps (mangals) are important marine‐influenced wetlands whose primary producers are overwhelmingly angiosperms of terrestrial origin. These highly productive ecosystems at the transition between land and sea enrich nearby marine habitats with nutrients and facilitate colonization of lineages from land to sea or vice versa. Both marine and terrestrial animals have become specialized for life on or under mangroves and saltmarsh vegetation.

Although comprehensive descriptions of these ecosystems have long been available (Chapman, 1960; Greb et al., 2006; Macnae, 1968; Saintilan, 2009; Visser et al., 2019; Walsh, 1974), and numerous taxonomic and ecological studies of resident taxa have been published, differences in the times of origin between mangals and saltmarshes have gone unnoticed. These are nonetheless important because they might indicate that temperate gains in productivity in coastal vegetations are much more recent than those in tropical mangals.

Mangals and saltmarshes have in common that both thrive on and create muddy or sandy soils in wave‐sheltered tidal environments. Nevertheless, their taxonomic compositions differ strikingly, potentially reflecting contrasting times and places of origin. Here, I propose the hypothesis that saltmarshes are much younger than mangals, that the plant inhabitants of saltmarshes derive from lineages almost entirely different from the lineages of mangrove species, and that specialization of molluscs to mangrove swamps and saltmarshes mirror these contrasting histories.

## 2 MATERIALS AND METHODS

Plants were considered to be adapted to mangals or saltmarshes if they are regularly or occasionally inundated by seawater. Molluscs were considered to be specialized to mangroves or saltmarshes if they either routinely climb on vegetation or are attached to vegetation. Many species live on the sediment beneath vegetation or are found on hard surfaces in addition to the vegetation; these were not considered to be specialized mangal or saltmarsh species.

Phylogenetic and fossil evidence for times of origin were gleaned from the published literature. I searched for relevant phylogenetic and paleobotanical papers for each family of angiosperms with representatives in saltmarshes and then consulted the reference list in the papers as well as the papers that cited those I found. Search terms other than family names were judged inadequate or too general to be useful. Divergence times were accepted as inferred in the studies cited. Given their consistency among papers, variations in the protocols used should have a little effect on the interpretations made herein.

## 3 RESULTS

### 3.1 Plants

At the family level, there is almost no overlap between plant lineages in mangals and those in saltmarshes. Among angiosperms, 15 families have representatives in mangals: Acanthaceae, Arecaceae, Combretaceae, Euphorbiaceae, Lythraceae, Malvaceae, Meliaceae, Myrsinaceae, Myrtaceae, Plumbaginaceae, Rhizophoraceae, Rubiaceae, Sapotaceae, Stercullaceae, and Tetrameristaceae. There are 11 families with species restricted to saltmarshes: Amaranthaceae, Asteraceae, Batidaceae, Caryophyllaceae, Frankeniaceae, Juncaceae, Juncaginaceae, Plantaginaceae, Plumbaginaceae, Poaceae (Chloridoideae and Pooideae), and Primulaceae. Specialization to these saline coastal environments has occurred more than once in most of these families (Bennett et al., 2013; Dassanayake & Larkin, 2017; Ellison et al., 1999; Flowers et al., 2010; Ricklefs & Latham, 1993; Sahu et al., 2016). Only one family (Plumbaginaceae) includes representatives in both habitats, but mangal and saltmarsh species belong to separate lineages. There is no genus‐level overlap between mangrove and saltmarsh plants. Three seagrass families, all belonging to Alismatales (Larkum et al., 2017; Les et al., 1997) contain no species in mangals or saltmarshes. In addition to angiosperms, the fern genus *Acrostichum* (Pteridaceae) has adapted to mangals (Wei et al., 2020), and several mosses tolerate or even have become restricted to saltmarshes (Adam, 1976; Callaghan & Farr, 2018; Garbary et al., 2008). Altogether, then, at least 26 family‐level groups of vascular plants occur in mangals or saltmarsh vegetation.

With the possible exception of the chloridoid grass genera *Distichils* and *Sporobolus*, which have warm‐temperate saltmarsh representatives but are otherwise tropical in distribution, saltmarsh plant lineages have temperate origins. Moreover, many prominent saltmarsh genera contain species or populations that live in other habitats, including inland saline areas as well as fully terrestrial settings. Examples include *Artemisia* and *Aster* (Asteraceae), *Juncus* (Juncaceae), *Plantago* (Plantaglnaceae), *Puccinellla* (Pooideae), *Spartina* (Chlorldoideae), *Spergularia* (Caryophyllaceae), and *Triglochin* (Juncaginaceae) (Gillespie et al., 2008; von Mering & Kadereit, 2015; Pimentel et al., 2017). All saltmarsh plants are low‐growing and have ancestors in open, non‐forested areas. Mangrove genera, by contrast, comprise mangal specialists and are trees with tropical‐forest origins (Sahu et al., 2016).

A second striking contrast between angiosperm‐dominated mangals and saltmarshes is the time of origin of these ecosystems. Mangals are first documented for the Maastrichtian (late Cretaceous) with the appearance of *Nypa* (Arecaceae), *Palaeowetherellia* (Euphorbiaceae), and the fern *Welchselia reticulate* (Greb et al., 2006; Ricklefs & Latham, 1993; Sahu et al., 2016; Shinaq & Bandel, 1998). *Rhizophora* (Rhizophoraceae) and *Pelliciera* (Tetrameristaceae) are known from the Paleocene, followed by *Acrostichum* and *Avicennia* (Acanthaceae) in the Early Eocene and *Lumnitzera* (Combretaceae) in the Middle Eocene, *Sonneratia* (Lythraceae) and *Camptostemon* (Malvaceae) in the Early Miocene, and *Excoecaria* (Euphorbiaceae) in the Middle Miocene (Ricklefs & Latham, 1993; Sahu et al., 2016). Mangal ecosystems have expanded and contracted throughout the Cenozoic in all parts of the tropics, but their highest diversity of plant species is achieved in the inner Indo‐West Pacific region of southeast Asia (Ellison et al., 1999; Guo et al., 2017; Walsh, 1974; Woodroffe & Grindrod, 1991).

The history of saltmarsh lineages is less well known, but divergence times inferred from molecular sequences indicate that saltmarshes are no older than the latest Eocene to earliest Oligocene (35–30 Ma). The grass subfamily Chloridoideae, which have C_4_ photosynthesis, is estimated to have originated about 35 Ma (Bouchenak‐Khelladi et al., 2014). Saltmarsh members of chloridoid genera such as *Distichlls*, *Spartina*, and *Sporobolus*, therefore cannot be older and are likely much younger, dating to the Middle or Late Miocene (Bouchenak‐Khelladi et al., 2014; Greb et al., 2006). The Salicornioideae (Amaranthaceae), with about 100 saltmarsh species worldwide, is estimated to have evolved in the Late Oligocene to Early Miocene (25–20 Ma) (Piirainen et al., 2017). This clade belongs to the order Caryophyllales, a clade with mid to late Cretaceous origins (Yao et al., 2019) and to which several other saltmarsh groups (Caryophyllaceae, Frankeniaceae, and Plumbaginaceae) belong. In the Asteraceae, another major clade with late Cretaceous origins (Mandel et al., 2019), tribes with representatives in saltmarshes began diversifying in the latest Eocene (36 Ma), indicating that this is a maximum age of saltmarsh members of the family (Mandel et al., 2019). The C_3_‐photosynthesizing grass subfamily Pooideae, which includes saltmarsh species of *Puccinellia*, also originated in the Late Eocene and diversified explosively in the Oligocene and Miocene (Pimentel et al., 2017). A similar scenario has been proposed for *Triglochin* (Juncaginaceae), which originated about 36 Ma. The saltmarsh *T*. *bublosa* and *T*. *marltlma* complexes have a crown age of 10 Ma (Late Miocene) (von Mering & Kadereit, 2015). In short, estimates of divergence times all point to a maximum age of latest Eocene for saltmarsh lineages, with most taxa likely being much younger. If these inferences are correct, the origin of a terrestrially derived saltmarsh vegetation would postdate the origin of mangal vegetation by at least 34 m.y. The latest Eocene origin coincides with the earliest Antarctic glaciation and with high‐latitude cooling in both hemispheres.

### 3.2 Marine molluscs

Animals of marine origin are highly diverse in mangals, especially in the Indo‐West Pacific. Among tree‐climbing gastropods, they include members of Cerithiidae (*Clypeomorus pellucida*), Littorinidae (*Littoraria*, *Littorinopsis*, and related genera), Muricidae (species of *Indothais* and *Thaisella*), Neritidae (*Ilynerita* and some *Cymostyla*), and Potamididae (*Cerithidea* and *Terebralia*) (Claremont et al., 2013; Reid et al., 2008, 2010; Vermeij, 1973). Many additional gastropods live on the soil beneath mangroves. Likewise, although many bivalves occur in mangrove swamps, most live on a variety of substrata. The only bivalves that appear to be specialized for attachment to mangroves belong to Anomiidae (*Enigmonia aenigmatica*, on leaves) and Isognomonidae (several species of *Isognomon*) (Tëmkin & Printrakoon, 2016; Yonge, 1957).

Species in at least 10 gastropod families live on or under vegetation in saltmarshes, but only one species, the northwest Atlantic *Littoraria irrorata*, occurs on saltmarsh plants (Reid et al., 2010). Many gastropods have populations in both mangals and saltmarshes, but again, most of these do not climb on vegetation. Plant‐climbing species of *Palustorina* (Littorinidae) in China and *Littorinopsis* in Australia live in saltmarshes but are primarily associated with tropical mangroves (Dong et al., 2015; Reid, 1986). No saltmarsh gastropods that can climb plants have temperate origins. Northern‐hemisphere populations of *Littorina* occur in saltmarshes, but they belong to species with very broad ecological distributions including wave‐swept rocky shores (Reid, 1996). The only bivalve that appears to be specialized for life in saltmarshes is the byssate semi‐infaunal mytilid mussel genus *Geukensia*, with two allopatric northwest Atlantic species (Sarver et al., 1992). *Geukensia* in the sister group of the tropical and warm‐temperate western Atlantic genus *Ischadium* in the subfamily Brachidontinae, which occurs widely in mangals and on other hard substrata including oysters (Trovant et al., 2015).

Mangrove‐associated littorinids and potamidids date to the Early Eocene (Dominici & Kowalke, 2014; Reid et al., 2008, 2010), but the tree‐climbing potamidid *Cerithidea*, *Clypeomorus* (Cerithiidae), and muricids are no older than the Miocene (see also Claremont et al., 2013; Houbrick, 1985). The saltmarsh‐specialized *Littoraria* irrorata is a known fossil from the Late Miocene and Pliocene and diverged from its sister species *L*. *varia* at about this time (Reid et al., 2010). The littorinids with well‐established saltmarsh populations in the North Atlantic (*Littorina littorea* and *L*. *saxatilis*) arrived from the North Pacific during the Pliocene (Reid, 1996). There is no pre‐Pleistocene record for the mussel genus *Geukensia*. The meager record of saltmarsh molluscs is therefore consistent with the hypothesis that saltmarsh ecosystems are much younger than mangals and that specialization to saltmarshes is much less common than that to mangroves.

## 4 GENERAL DISCUSSION

If the hypothesis that angiosperm‐dominated saltmarshes are much younger than mangals is correct, as phylogenetic and fossil evidence from plants and molluscs indicate, it would have far‐reaching implications for ecological and evolutionary connections between terrestrial and nearby coastal marine ecosystems. Both saltmarshes and mangals are highly productive ecosystems that facilitate nutrients exchange by mobile animals between land and sea. Pre‐angiosperm halophytes may have existed as well, but these were likely less productive and less diverse. Successions of mangal‐like communities might have existed during the Carboniferous, but the evidence for specialized tidal saline vegetation during the Paleozoic is suspect (Falcon‐Lang, 2005; Greb et al., 2006). The peculiar lycophyte *Pleuromeia* from the Early and Middle Triassic appears to be halophytic (Krassilove & Zakharov, 1975; Retallack, 1975), as are some conifers of the Middle Jurassic and Early Cretaceous in the family Cheirolepidiaceae, the Middle Jurassic conifer *Brachyphyllus*, and the Late Jurassic fern *Pachypteris* (Alvin, 1982). The taxonomic affinities of these taxa indicate low productivity in comparison with angiosperms. Before angiosperm mangroves evolved in the Late Cretaceous, sheltered shores in warm regions would have been less hospitable to both marine and terrestrial animals. Once these environments were vegetated by angiosperms, the transitional tidal systems accommodated more animal life and physically less harsh conditions. Low‐diversity mangrove assemblages extended north to Arctic latitudes during the warmest phases of the Eocene, but mangroves retreated southward (or northward, depending on the hemisphere) as climates cooled thereafter (Popescu et al., 2021), leaving temperate mudflats potentially unvegetated.

High‐latitude cooling began about 35 Ma and intensified about 13 Ma during the middle Miocene. Habitats available for colonization by saltmarsh plants therefore expanded from non‐forested temperate regions. Unlike the plants, the small number of saltmarsh‐specialized molluscs has tropical origins. Molluscs with broad habitat distributions have opportunistically colonized saltmarshes from either tropical or temperate populations.

The hypothesis proposed here can be further tested and elaborated by considering other animal groups that have become specialized to live on mangrove trees or in saltmarshes. These groups especially include brachyuran crabs as well as herbivorous insects and some terrestrially derived vertebrates. Geographical differences in specialization and in diversity of mangal and saltmarsh specialists could illuminate aspects of evolution in these habitats. For example, Indo‐West Pacific mangrove plants, molluscs, and crustaceans are more than 10 times richer in species than their Atlantic‐East Pacific counterparts (Ellison et al., 1999; Vermeij, 1973; Walsh, 1974), perhaps reflecting the geographical extents of suitable habitats in the past (Ricklefs & Latham, 1993). Differences in saltmarsh specialization between regions have not been investigated, but the northwest Atlantic and perhaps Australia appear to harbor more specialists than other parts of the world.

These historical and comparative aspects of ecology can be fruitfully applied to other ecosystems as well, especially systems that develop at major transitions among terrestrial, freshwater, and marine environments. Although such studies may not have immediate practical applications, they offer an important historical perspective with long‐term implications for the health and sustenance of systems that have been strongly affected.

## CONFLICT OF INTEREST

I wrote the paper, and I have no conflicts of interest.

## AUTHOR CONTRIBUTIONS

**Geerat Vermeij:** Conceptualization (lead); Investigation (lead); Writing – original draft (lead).
